# Complications of Preterm Birth—The Importance of Care for the Outcome: A Narrative Review

**DOI:** 10.3390/medicina60061014

**Published:** 2024-06-20

**Authors:** Jelica Zivaljevic, Miljana Z. Jovandaric, Sandra Babic, Misela Raus

**Affiliations:** 1 Department of Neonatology, Clinic for Gynecology and Obstetrics, University Clinical Center of Serbia, 11000 Belgrade, Serbia; 2 Department of Gynecology and Obstetrics, Clinic for Gynecology and Obstetrics, University Clinical Center of Serbia, 11000 Belgrade, Serbia; 3 Department of Neonatology, University Children’s Hospital, 11000 Belgrade, Serbia; 4 Faculty of Medicine, University of Belgrade, 11000 Belgrade, Serbia

**Keywords:** preterm newborn, modern therapy, presence of parents, complications of preterm birth

## Abstract

Preterm-born children are susceptible to problems of adaptation in the early neonatal period, as well as the emergence of consequences due to the immaturity of the respiratory, cardiovascular, and especially cerebrovascular systems. The authors searched PubMed, Scopus, the Cochrane Library, and Web of Science for articles that were available in their entirety and published in English between 1990 and 2024 in peer-reviewed journals using keywords relevant to the manuscript topic. Analyzing the requested studies and manuscripts, adequate articles describing the stated problem were used. The last trimester of pregnancy is the most important period in brain development. Brain growth is at its most intense, and nerve cells are created, multiply, and migrate, creating numerous connections between them and receptors. During this period, the baby is protected from the influence of external environmental factors. When a baby is born, it leaves its protected environment and very often requires intensive treatment to survive. In these circumstances, the immature nervous system, which is in a sensitive stage of development, is overloaded with numerous external stimuli, continuous light, noise, inappropriate positioning, and repeated painful reactions due to necessary diagnostic and therapeutic procedures and the unavoidable absence of the mother and the family, which cause stress that threatens proper programmed development. Minimally invasive therapeutic procedures and the presence of parents during hospitalization play a significant role in reducing the consequences for a premature child.

## 1. Introduction

Preterm birth has been one of the biggest public health problems in recent decades. The increased percentage of survival represents a great challenge for perinatal and neonatal medicine to understand and prevent the neurodevelopmental consequences for preterm children [1,2]. The first neonatal intensive care unit was established in the United States in 1965 at the Yale University School of Medicine. In 1980, exogenous surfactant was used for the first time in the treatment of respiratory distress syndrome [3,4]. The next advance in perinatal medicine was the antenatal use of steroids, following the 1994 New York Declaration [5]. The use of surfactants and antenatal corticosteroids were revolutionary therapies that have increased the survival of preterm babies [6].

The application of new knowledge in clinical practice, as well as the development of technology in the form of ventilators with different ways of offering respiratory support, and knowledge of the nutritional needs of newborns significantly improved outcomes and reduced the consequences for preterm children. Adequate therapy and care in the first hours, days, and weeks, as well as a multidisciplinary approach by a team of experts from various specialties, are key factors for a favorable outcome for and further progress of preterm children. During the long-term follow-up of preterm children, various complications can be observed that can affect the quality of life [7].

It is widely recognized that preterm birth is associated with an increased risk of long-term health and neurodevelopmental problems [8]. The advantages of mother’s milk and early breastfeeding are significant for the prevention of necrotizing enterocolitis and also play an important role in the development of cognitive abilities in childhood, as they affect the structural development of the brain by increasing the white matter of the brain and the thickness of the cortex. Human breast milk components thought to mediate improved cognitive outcomes include long-chain polyunsaturated fatty acids and oligosaccharides [9]. Noise, pain, and the neonatal intensive care environment have been found to affect the neurodevelopment of infants, particularly as noise and pain have adverse effects. Isolated intensive care rooms do not have priority over open rooms [10]. Preterm babies in the neonatal intensive care unit (NICU) are exposed to stress from high-intensity tones. Hearing impairment is diagnosed in 2% to 10% of preterm infants compared to 0.1% of the general pediatric population. Noise can cause apnea, hypoxemia, and increased oxygen consumption due to increased heart rate and breathing, and therefore can reduce the amount of calories available for growth [11]. Research on the impact of NICU pain exposure on functional brain development is relatively limited; however, greater exposure to pain in the NICU, due to various interventions, is associated with various health and neurodevelopmental changes, including reduced infant weight, lower white matter fractional anisotropy, and lower school-age IQ, as well as reduced cerebellar volume in school years [12]. Exposure to early life pain (ELP) is associated with a low pain threshold in adulthood. Underlying ELP are mechanisms that include changes in peripheral skin innervation, peripheral afferent sensitization of spinal cord nociceptive circuits, and altered descending pain control in the brainstem [13]. The presence of parents in the neonatal intensive care unit (NICU) affects weight gain and decreases the rate of infection in newborns [14].

Preterm birth is defined as any birth before the 37th week of gestation. About 15 million babies are born preterm each year (5% to 18% of all births). This rate varies from country to country. In the United Kingdom, 7.9% of babies are born preterm, and in the United States, 12.3% of all births take place before 37 weeks of gestation [15]. About 0.5% of babies are born between 20 and 25 weeks of gestation and represent the highest percentage of infant mortality [16]. The chance of survival in the 22nd week is about 6%, while in the 23rd week it is 26%, in the 24th week it is 55%, and in the 25th week it is about 72%. The chances of survival without long-term difficulties are lower [17]. With the introduction of new treatment measures, more than 95% of preterm babies survive [18].

## 2. Material and Methods

The authors searched for available data on preterm birth, the impact of preterm birth on complications and neonatal outcomes, as well as the importance of care and parental presence for the prevention of complications of preterm birth. The authors searched PubMed, Scopus, the Cochrane Library, and Web of Science for articles available in their entirety and published in English between 1990 and 2024 in peer-reviewed journals. To search the literature, we used a combination of keywords: “preterm birth”, “complications of preterm birth”, “newborn”, “care”, “the role of parents in the care of the newborn in the NICU”, “breastfeeding “, “the influence of external environmental factors in the NICU”, “octopus”, “outcome”, and “treatment”. Articles and studies related to the treatment of preterm infants, as well as manuscripts whose full texts were not available or which were not written in English, were excluded from the study. For this narrative review, the manuscripts considered by the authors to be the most important for the topic were selected.

The findings of the literature search are presented in Section 3 and Section 4, together with a discussion illustrating the current evidence on the importance of care in the prevention of complications in premature infants; the elimination of environmental factors in the NICU in the form of noise, light, and pain; and the presence of parents during the NICU stay for neonatal outcomes.

## 3. Complications of Preterm Birth

Newborns born before the end of term gestation face many challenges to their survival in extrauterine conditions. Preterm children are not only at high risk of death but also at increased risk of long-term neurological disability, impaired language development, reduced cognitive abilities, and greater risk of cardiovascular disease and diabetes. Furthermore, multi-organ immaturity has resulted in respiratory distress, interventricular hemorrhage, sepsis, thrombocytopenia, blindness, and gastrointestinal disturbances [10].

### 3.1. Early Neonatal Complications of Preterm Neonates 

Early complications are most often manifested as respiratory distress syndrome (RDS), patent ductus arteriosus (PDA), early sepsis, necrotizing enterocolitis (NEC), and intraventricular hemorrhage (IVH) [19].

The cause of RDS is the immaturity of the lungs or a disorder in the synthesis and function of surfactant due to pathological processes (asphyxia, congenital pneumonia, and pregnancy diseases) [20].

The fetal ductus arteriosus (DA) diverts cardiac output from the lungs to the placenta to support systemic oxygenation. Increased pulmonary blood flow in premature infants can lead to pulmonary edema; worsened breathing; and reduced gastrointestinal, renal, and cerebral blood flow. The incidence of PDA (i.e., open DA after the first 3 postnatal days) exceeds 50% in preterm infants born at ≤28 weeks of gestation [21,22]. The incidence of early neonatal sepsis (<3 days of life) ranges from 1 to 5 per 1000 live births [23].

Necrotizing enterocolitis (NEC) is a leading cause of morbidity and mortality in premature infants. A characteristic of NEC is the rapid development of necrosis of the intestinal wall with perforation and secondary peritonitis. Even with early therapy, the prognosis is poor, with a mortality of 15% to 30% [24].

The most common neurological complication is intraventricular hemorrhage (IVH). It is seen in the germinal matrix, where capillaries are immature, vascularization is intense, and active cell proliferation is high [25].

### 3.2. Late Complications of Preterm Birth 

Despite advances in treatment, with increased survival rates, the number of children with irreversible visual impairment has increased. It is estimated that around 32,300 infants have irreversible visual impairment due to the development of retinopathy of prematurity (ROP) [26,27].

Bronchopulmonary dysplasia (BPD) is a chronic lung disease that primarily affects preterm infants who receive respiratory support with either mechanical ventilation or supplemental oxygen. The incidence of BPD in infants born at 28 weeks of gestation is 23%, of whom 8% develop severe BPD [28].

Poor long-term outcomes (cerebral palsy and intellectual disability) are associated with the occurrence of periventricular leukomalacia (PVL). Although not a direct consequence of IVH, PVL is a condition often associated with bleeding and reflects selective necrosis of the periventricular white matter [29].

Late neonatal sepsis occurs in 0.61% and 14.2% of hospitalized infants [30]. It is caused by the contact of newborns with medical staff. The most common causative agent is coagulase-negative staphylococcus (53.7–77.9%), although other causative agents, such as *Staphylococcus aureus*, *Candida* spp., *Klebsiella* spp., *Pseudomonas* spp., *Escherichia coli*, and Enterobacters, can also be found [31,32]. It appears after the third day of life and is a cause of high mortality rates, weaker neurodevelopment, and slower growth [33].

Preterm birth is associated with reduced nephron number and kidney size. Most nephrons are formed in the third trimester of pregnancy, between the 28th and 34th weeks of gestation. Fewer nephrons are associated with the development of hypertension and the risk of chronic kidney disease later in life [34].

In addition to kidney disease, preterm neonates are prone to developing diabetes in adulthood. Intrauterine nutritional abnormalities can also permanently alter metabolism, leading to early fetal programming for the future development of diabetes and other cardiometabolic disorders [35].

It was shown that, even at a later age, preterm neonates do not achieve the same results as their peers born at term, from which it was concluded that this effect is not a matter of intellectual disability but of a deficit in general functions and intelligence. Another negative predictor of cognitive development refers to gender, where a significant difference was found in favor of girls, who showed better results in cognitive abilities. A possible explanation lies in the changed hormonal status of preterm children and the increased testosterone concentration in male children, which increases the risk of early brain damage [36].

## 4. Care and Treatment of a Preterm Child

Care for preterm newborns is the most important factor for preventing mortality and mitigating the consequences of morbidity. Preterm-born children are admitted to neonatal intensive care units and later continue treatment in neonatal intermediate units. A child born at term has developed all his senses—he is ready to interact with the outside world. Interaction is more difficult for a preterm baby—the environment with its loud sounds and bright lighting hurts him. As a result, the baby may later have hearing, vision, and sleep disorders. Therefore, in hospital conditions, continuous lighting is abolished, the lights are turned off when the clinical condition of the baby allows it, the incubators are covered with special blankets to enable longer periods of peaceful sleep and rest, and immature eyes are dosed with the optimal amount of light for the stage of development [37].

Reducing the sound levels reaching newborns to 45 dB or less can be achieved by reducing sound levels throughout the unit; treating babies in the NICU, in a “private” room, or in incubators where sound levels are controlled; or by reducing sound levels that reach individual babies through the use of earmuffs or earplugs. By reducing the level of sound reaching newborns, the impact of stress on the cardiovascular, respiratory, neurological, and endocrine systems is reduced, thus promoting growth and reducing adverse neonatal outcomes [11].

The Newborn Individualized Developmental Care and Assessment Program (NIDCAP) is a special program for access to preterm newborns. It is a specific method of care and assessment of newborn behavior; it is also an early intervention program, individually adapted to each baby and family-oriented, which means that its implementation actively involves family [38]. Preterm newborns need a special individual approach to treatment and care, which is provided first in the intensive care unit for newborns and continues in the department of pathology and in the care of small newborns. Babies should be in conditions as similar as possible to the intrauterine environment. Very premature babies are placed in special incubators where temperature, humidity, light intensity, and sound volume are controlled and the necessary support for all vital functions (nutritional, respiratory, and hemodynamic) is provided. Those with a higher body weight are accommodated in beds equipped with special heated mattresses. A preterm baby cannot control its body, so if it finds itself in a stretched position, due to the impossibility of coordinating movements, that position means disorganization for the baby. For the baby to take a position as close as possible to the intrauterine, special rollers, so-called nesting boxes, are used. The arms and legs are arranged in a bent position, pressed against the body, ensuring less movement to save energy [39].

Given that tactile contact can be painful for a preterm baby, the number of interventions and manipulations should be minimized. Maternal contact has physiological benefits, such as the preservation of warmth and the emotional benefits of mental bonding and relaxation [40]. Active parental involvement in the NICU through close skin-to-skin contact with both parents of the baby is associated with improved neurobehavioral outcomes at 4 to 5 years of age. In addition, it reduces stress and pain in the newborn, and the frequent presence of and holding by parents can also lead to stronger parent–child attachment, which forms the basis for later interactions that can improve the child’s development; in this way, the risk of attachment disorders that are characteristic of premature children is reduced [14].

Cuddling and contact with the mother’s chest should be provided even if the baby is on mechanical ventilation. For premature babies, there are mechanical ventilation systems that allow babies to breathe until their lungs and the respiratory center in the brain matures [41].

On the wards, children are connected to special electronic monitors that monitor their heart rate, blood oxygen saturation level, and breathing rate, which reflect their general condition and level of maturity. At the slightest sign of a change in the parameters of vital functions, an alarm is immediately sounded, and the nurse checks the connection of the pulse oximeter and the correctness of the equipment and keeps a record of the change in the child’s condition, that is, the entire staff team immediately takes the necessary actions to normalize the baby’s condition. Intravenous lines, umbilical vein catheters, and umbilical artery catheters can save the life of an extremely premature baby. If the child does not have sucking and swallowing reflexes, feeding is performed with an orogstric tube, and, as a last resort, a stomach tube is placed in the child. Before that, she will be given breast milk or special formulas for preterm babies until she can feed herself. In many intensive care units, children are in constant contact with their parents, which is especially important when caring for preterm babies. It is the contact of the baby’s naked skin with the skin of the mother or father that is significant [42].

**Figure 1:**
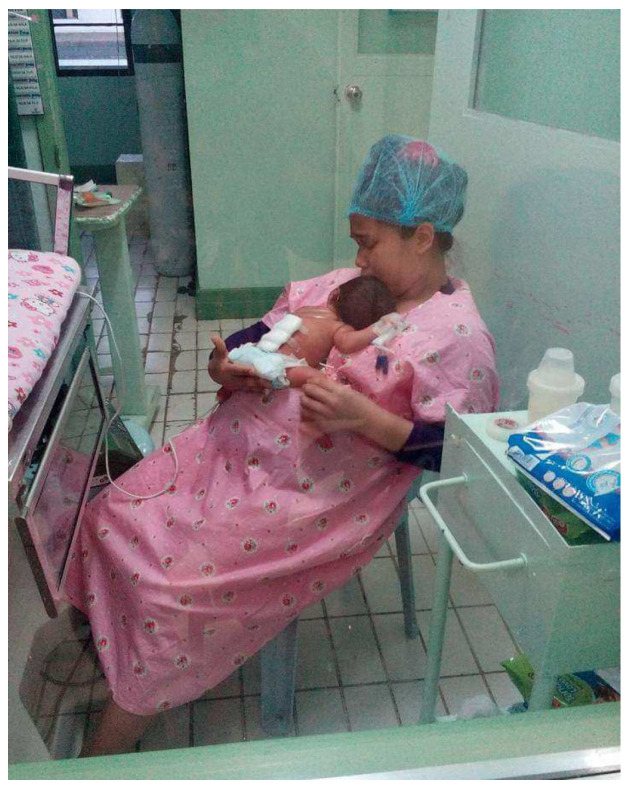
Contact with mother (the “kangaroo care” method). In contact with the mother, this method has a calming effect, relaxes the baby, and stimulates the secretion of hormones in the mother and the production of milk. The baby has better body temperature regulation, breathes more regularly, has a better gas exchange, and the heart works more regularly. Bacteria from the mother’s skin and breast colonize the baby’s intestinal tract, which promotes proper functioning of the intestines and better immunity, and thus reduces consequences of early birth.

Preterm babies are placed in special devices—“kangaroos”—in which the parents carry them on themselves. The “kangaroo” method implies close contact between the baby and the mother—“skin to skin”. The father can also participate in the process and replace the mother in case of illness or ill health. With gentle movements, the nurse opens the incubator door and teaches the mother how to properly hold the baby, or, in case of fear, puts the baby on the mother’s chest. The basic idea of the method is to lay the baby naked on the skin of the mother’s breast for several hours every day. The child is placed in a lying position with the face turned towards the mother, which resembles the “frog” position. To maintain temperature, a hat is placed on the baby’s head, and a warm blanket is placed on top. In the first days, the child is placed on the mother’s breast twice a day for 20–40 min. Then, the duration of the “session” gradually increases to several hours. After being discharged, the method can continue to be used at home. It has been proven that the “kangaroo” method not only warms the baby but also has a positive effect on its physiology and psychomotor development [43] (Figure 1).

The positive impact of this method is reduced consumption of the baby’s energy to create heat and reduced crying. Sleep and wakefulness are normalized. It improves breathing and heart rate, as well as oxygen saturation of the blood. The proximity of the mother’s breast and the smell of milk contribute to the development and coordination of innate reflexes: sucking, swallowing, and seeking. Maturation of the cerebral cortex is accelerated, allowing rapid recovery, as well as other psychomotor developments. The “kangaroo” method has proven to be the best in caring for a premature child, but it can be fully applied only after the child is completely stabilized, in the absence of convulsions, and after stabilization of the main parameters (breathing, heart rate, etc.) [44].

Nurses monitor the newborn’s condition, the operation of monitors, the introduction of drugs, nutrition, and care. The midwife in the maternity ward monitors the condition of the mother after giving birth, then she will continue to help the mother breastfeed the baby. If the baby is born too small to breastfeed, the pediatric nurse will train the mother how to properly breastfeed and deliver milk to the newborn. Nurses in the intensive care unit must fully provide all types of professional assistance to mothers of seriously ill children or premature babies. Thanks to modern technologies, significant success has been achieved in the care and survival of such children who have extremely low body weight. The most important factors for a good outcome for a preterm child are the gestational age at which the premature birth occurred as well as delivery in a health facility with optimal conditions for providing qualified medical care starting from the birth of the child. Prevention of hypothermia in preterm infants is an important care measure because their immature temperature regulation can lead to consequences such as apnea, hypoglycemia, and poor weight gain [45].

Noninvasive ventilation, especially the use of continuous positive airway pressure (CPAP), is essential in the management of preterm infants with respiratory problems. Due to the development of respiratory distress syndrome, it is necessary to use a surfactant. The methods of application differ, and it is preferable to apply the surfactant with the least invasive method because research has shown that both types of methods (with and without intubation of the newborn) are equally effective as standard treatments in terms of avoiding mechanical ventilation and reducing consequences, such as intracerebral hemorrhages and bronchopulmonary dysplasia [46].

After birth, all children receive vitamin K, which is involved in the production of prothrombin (a protein in the blood), for the prevention of blood clotting. When bleeding occurs, vitamin K is prescribed for three days. Baby skin massage has a special place in care. Special attention should be paid to the time of the start of massage because there is a risk of developing retinopathy of prematurity. Usually, the first massage sessions start at 1–1.5 months of life. Preterm babies often have disorders of muscle tone, which can be increased or decreased. With a raised tone, only light caressing is allowed, and with a reduced tone, rubbing, kneading, and tapping are performed. In this phase, the massage is combined with passive gymnastics: bending the arms and legs and turning the head. In the care of a preterm child, it is important to avoid noise, strong disorders, aggressive movements when opening the incubator, and loud speech. Incubators are covered to protect them from light. If the child needs phototherapy due to elevated bilirubin values, the eyes must be protected. A preterm baby is cared for by a team of specialists—a neurologist, an ophthalmologist, an endocrinologist, a pulmonologist, and often a surgeon—to correct complications of premature birth. When transporting premature babies, incubators, ventilators, infusion pumps, and devices for measuring vital parameters are used. Usually, the child is discharged home when he shows a positive trend, his weight reaches 2400–2500 g, and he can suck by himself or use a bottle. Apnea (cessation of breathing for more than 20 s) is common in immature children and sometimes lasts for months, even after returning home. In such cases, parents are required to obtain a special apnea monitor which turns on an alarm when the baby stops breathing. Any stimulation will help keep breathing going. Apnea monitoring is usually not necessary after six months, and the physiological slowing of breathing that comes with age should be taken into account. Other adaptation problems (which can be numerous) should have been overcome while the baby was in the hospital, so they are not expected to cause health problems after coming home [47]. 

**Figure 2:**
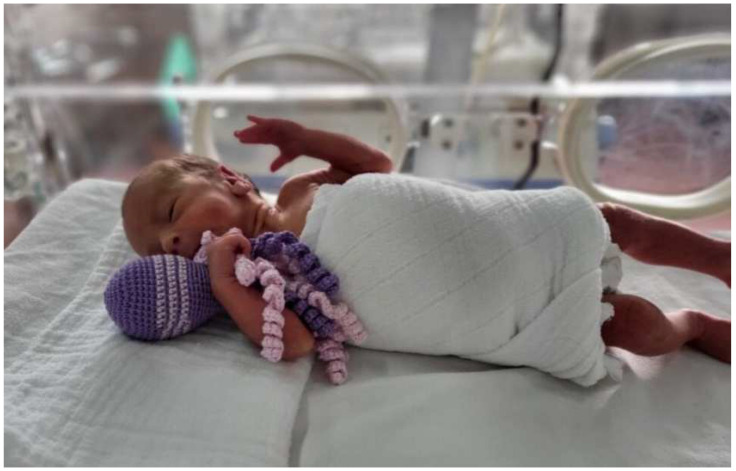
Octopuses. Octopuses are medical aids that are crocheted from a special thread suitable for maintenance and sterilization in hospital conditions that imitate the umbilical cord. While the newborn is in the stomach, it often touches the umbilical cord and thus develops contact with the mother. In the incubator, octopuses are placed on the baby’s skin for tactile stimulation, calmness, and stability of breathing and pulse. In addition, by grasping the arm of the octopus, babies do not grab feeding tubes or other tubes (e.g., umbilical catheters).

All over the world, octopuses are recognized as medical aids, which is why there is a specific procedure for making them. A mother’s hug cannot replace anything, and in that first phase of life, when tactile stimulation is especially pronounced, it is precisely the mother’s touch and hug that is most missed. Since preterm-born children are forced to be in an incubator and separated from their mothers, the octopuses somehow change their role. On the one hand, our little patients have what they need, and, on the other hand, octopuses make the work of medical professionals easier, since the baby does not feel the need to grab or tear the probes, feeding tubes, and other tubes which are there to ensure their progress in the incubator, as can happen when they do not have an octopus next to themselves. Octopuses soothe small patients and enable them to have a more stable state of health conditioned by basal functions such as heart rate and breathing [48] (Figure 2).

Octopuses are crocheted from a special thread suitable for maintenance and sterilization in hospital conditions. The crocheting method is characteristic, and it must be very dense so that bacteria cannot remain in the cavities. The dimensions are strictly determined—the head of the octopus has a diameter of 15–17 cm, and the length of the arm must not be longer than 22 cm—to imitate the umbilical cord, which the baby, while still in the stomach, often needs to touch and develop physical contact with the mother [49].

In general, it can be said that the advantages of the individual approach to each newborn are numerous, including a shorter period of invasive methods during the application of mechanical ventilation, fewer hours of oxygen therapy, reduced frequency of infections, reduced development of pulmonary complications, reduced frequency of brain hemorrhages, and therefore a reduced number of days of hospitalization; better psychomotor development measured at the ages of 3, 6, and 9 months; and better interaction between parent and child [50]. 

## 5. Conclusions

Modern therapeutic methods of treatment and minimally invasive procedures, along with the presence of parents, contribute to a quick recovery and reduced early and late consequences for preterm children.

## Data Availability

All data are available in the archives (databases) of medline and PubMed.
